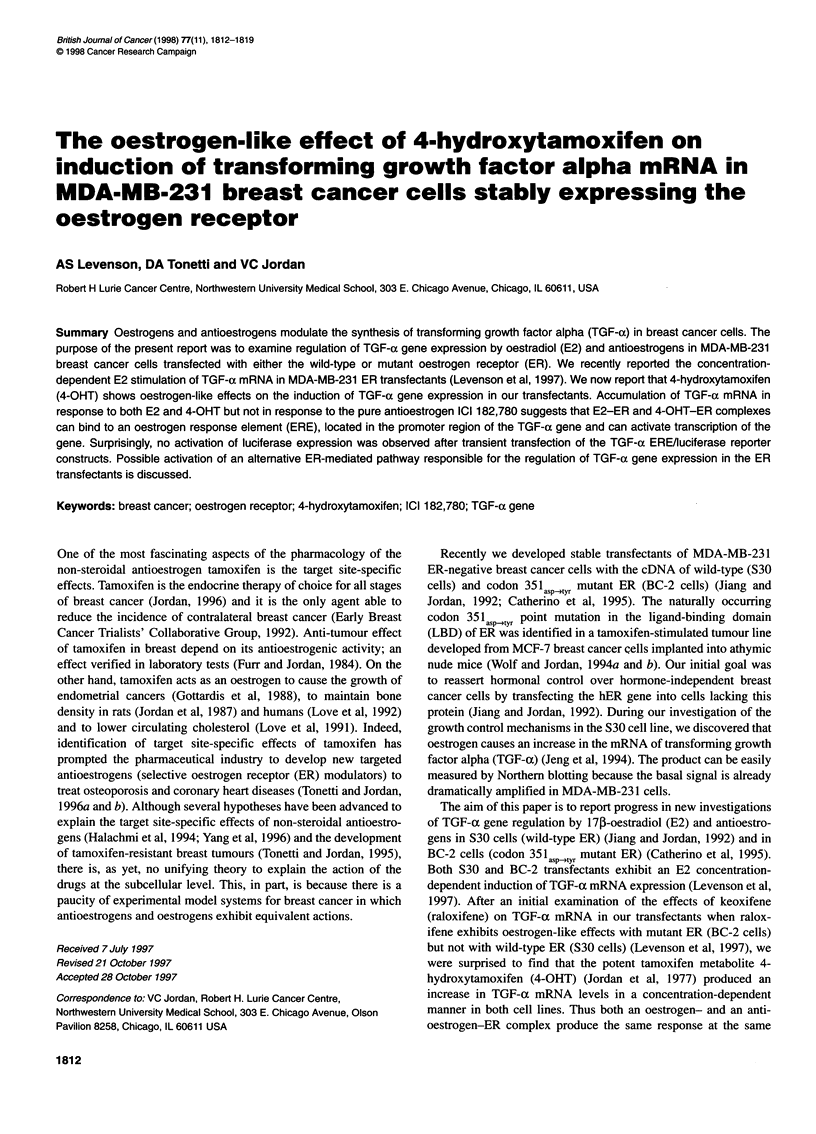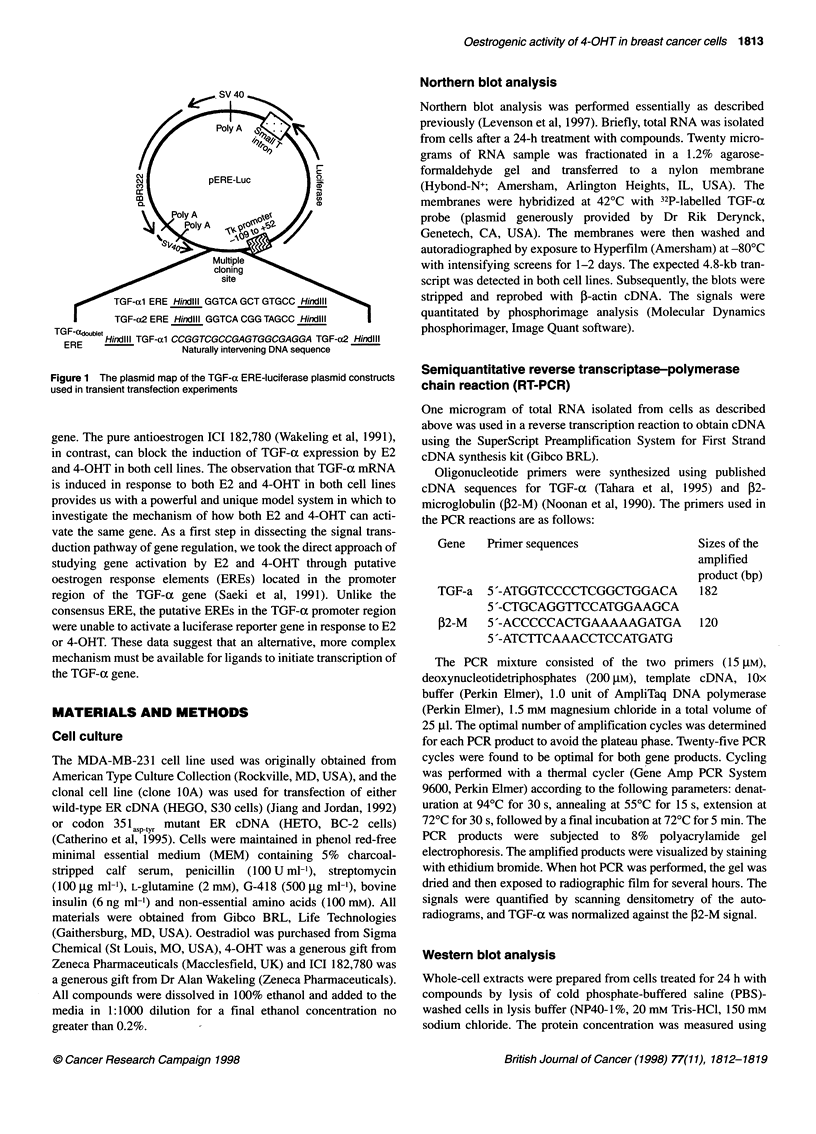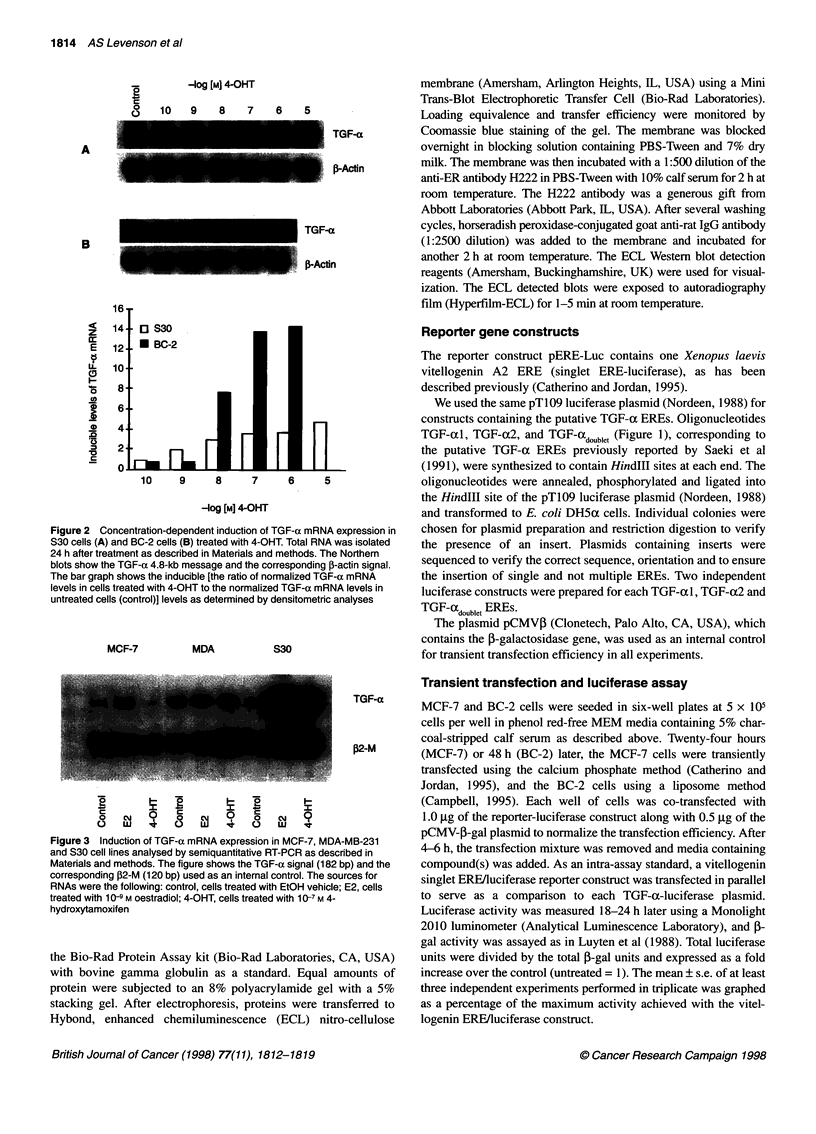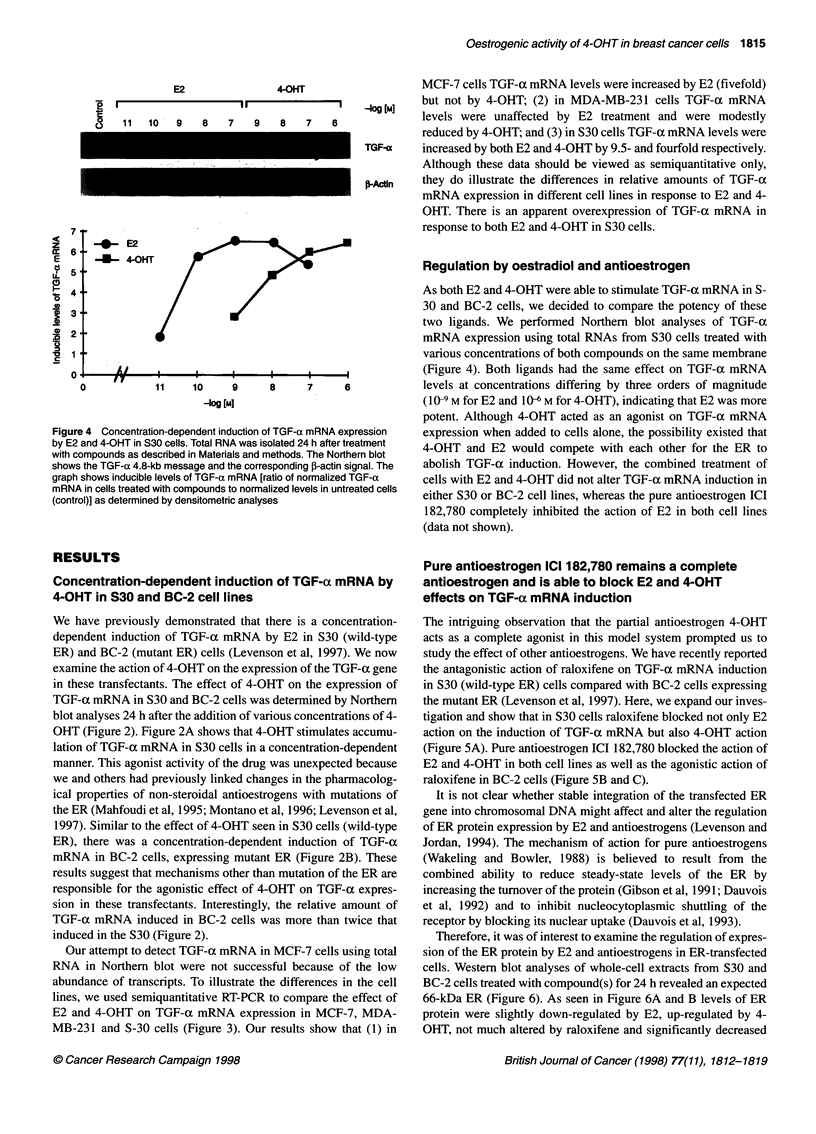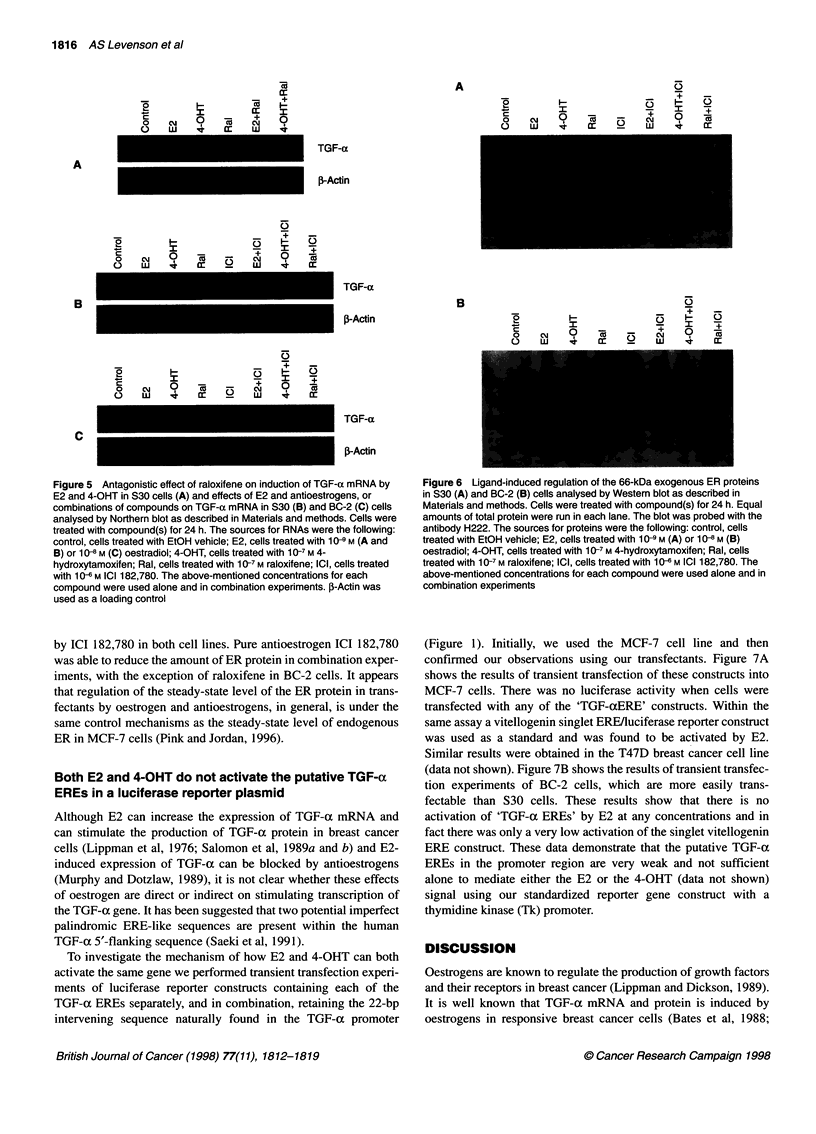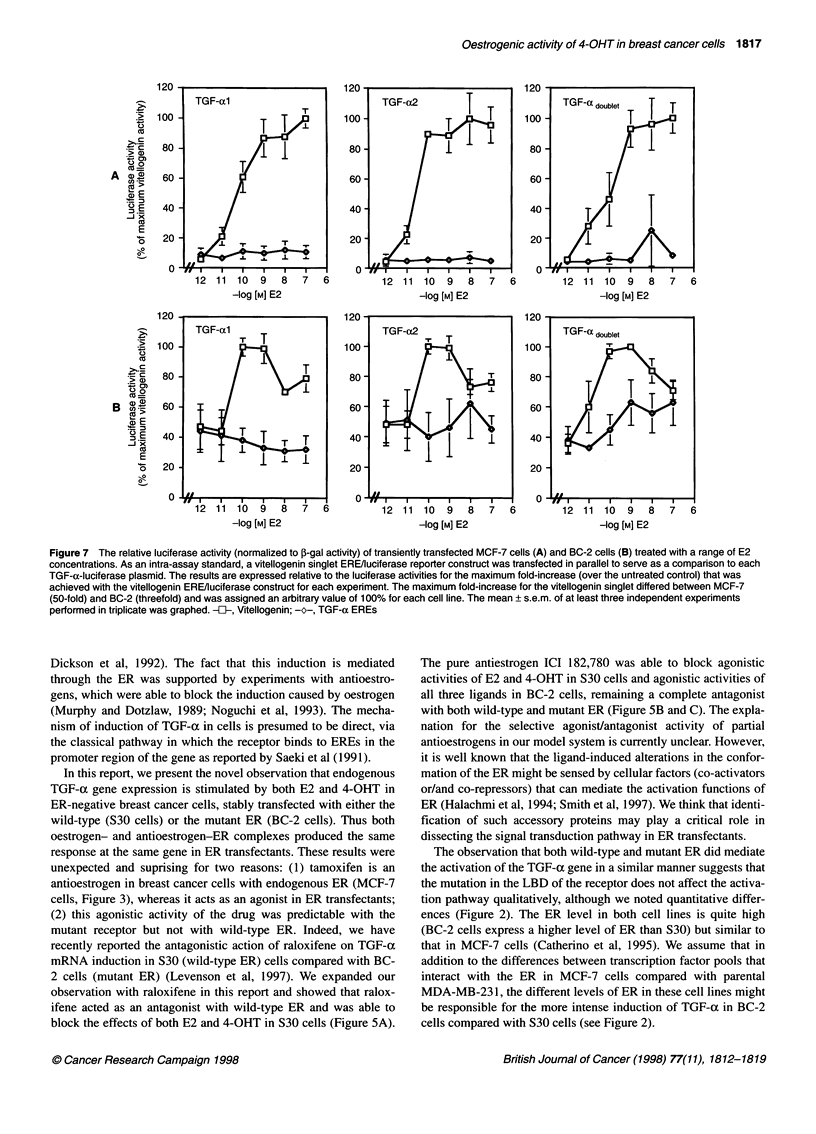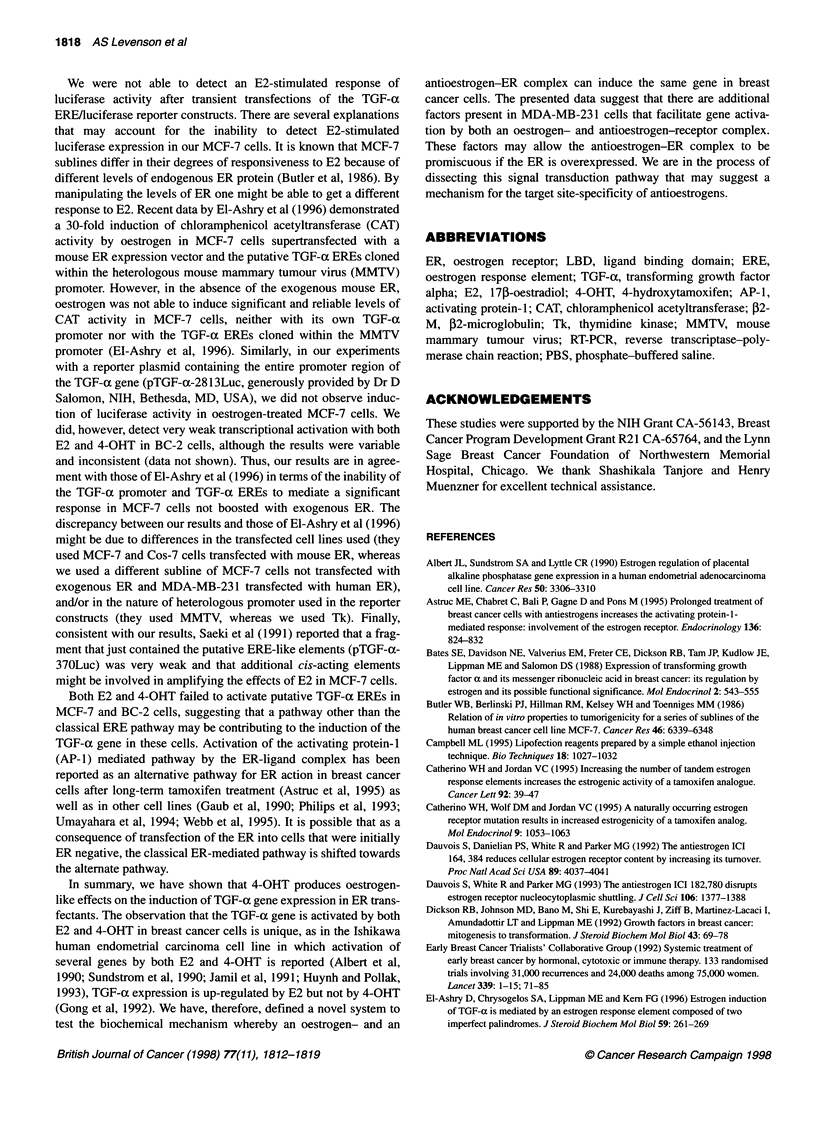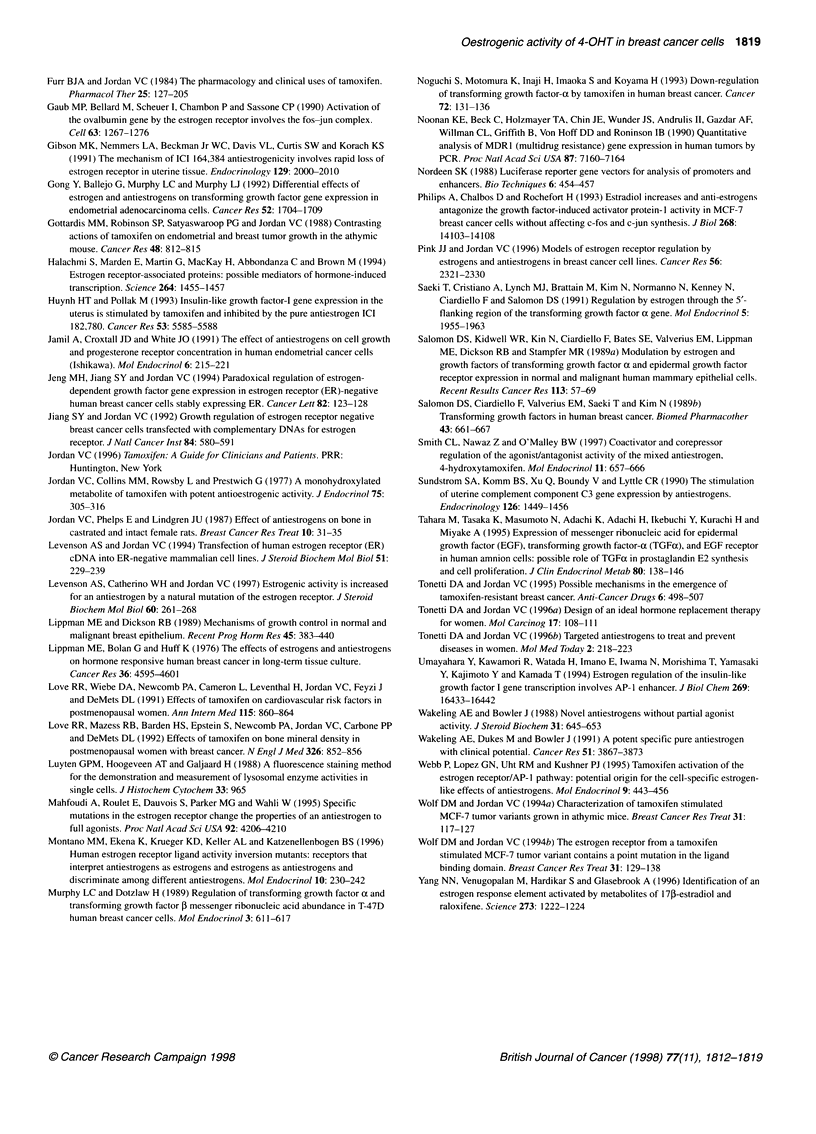# The oestrogen-like effect of 4-hydroxytamoxifen on induction of transforming growth factor alpha mRNA in MDA-MB-231 breast cancer cells stably expressing the oestrogen receptor.

**DOI:** 10.1038/bjc.1998.301

**Published:** 1998-06

**Authors:** A. S. Levenson, D. A. Tonetti, V. C. Jordan

**Affiliations:** Robert H Lurie Cancer Centre, Northwestern University Medical School, Chicago, IL 60611, USA.

## Abstract

**Images:**


*British Journal of Cancer* (1998) **77**(11), 1812–1819
© 1998 Cancer Research Campaign

### The oestrogen-like effect of 4-hydroxytamoxifen on induction of transforming growth factor alpha mRNA in MDA-MB-231 breast cancer cells stably expressing the oestrogen receptor

**AS Levenson, DA Tonetti and VC Jordan**

Robert H Lurie Cancer Centre, Northwestern University Medical School, 303 E. Chicago Avenue, Chicago, IL 60611, USA

**Summary** Oestrogens and antioestrogens modulate the synthesis of transforming growth factor alpha (TGF-α) in breast cancer cells. The purpose of the present report was to examine regulation of TGF-α gene expression by oestradiol (E2) and antioestrogens in MDA-MB-231 breast cancer cells transfected with either the wild-type or mutant oestrogen receptor (ER). We recently reported the concentration-dependent E2 stimulation of TGF-α mRNA in MDA-MB-231 ER transfectants (Levenson et al, 1997). We now report that 4-hydroxytamoxifen (4-OHT) shows oestrogen-like effects on the induction of TGF-α gene expression in our transfectants. Accumulation of TGF-α mRNA in response to both E2 and 4-OHT but not in response to the pure antioestrogen ICI 182,780 suggests that E2–ER and 4-OHT–ER complexes can bind to an oestrogen response element (ERE), located in the promoter region of the TGF-α gene and can activate transcription of the gene. Surprisingly, no activation of luciferase expression was observed after transient transfection of the TGF-α ERE/luciferase reporter constructs. Possible activation of an alternative ER-mediated pathway responsible for the regulation of TGF-α gene expression in the ER transfectants is discussed.

**Keywords:** breast cancer; oestrogen receptor; 4-hydroxytamoxifen; ICI 182,780; TGF-α gene

One of the most fascinating aspects of the pharmacology of the non-steroidal antioestrogen tamoxifen is the target site-specific effects. Tamoxifen is the endocrine therapy of choice for all stages of breast cancer (Jordan, 1996) and it is the only agent able to reduce the incidence of contralateral breast cancer (Early Breast Cancer Trialists' Collaborative Group, 1992). Anti-tumour effect of tamoxifen in breast depend on its antioestrogenic activity; an effect verified in laboratory tests (Furr and Jordan, 1984). On the other hand, tamoxifen acts as an oestrogen to cause the growth of endometrial cancers (Gottardis et al, 1988), to maintain bone density in rats (Jordan et al, 1987) and humans (Love et al, 1992) and to lower circulating cholesterol (Love et al, 1991). Indeed, identification of target site-specific effects of tamoxifen has prompted the pharmaceutical industry to develop new targeted antioestrogens (selective oestrogen receptor (ER) modulators) to treat osteoporosis and coronary heart diseases (Tonetti and Jordan, 1996*a* and *b*). Although several hypotheses have been advanced to explain the target site-specific effects of non-steroidal antioestrogens (Halachmi et al, 1994; Yang et al, 1996) and the development of tamoxifen-resistant breast tumours (Tonetti and Jordan, 1995), there is, as yet, no unifying theory to explain the action of the drugs at the subcellular level. This, in part, is because there is a paucity of experimental model systems for breast cancer in which antioestrogens and oestrogens exhibit equivalent actions.

*Received 7 July 1997*
*Revised 21 October 1997*
*Accepted 28 October 1997*

*Correspondence to:* VC Jordan, Robert H. Lurie Cancer Centre, Northwestern University Medical School, 303 E. Chicago Avenue, Olson Pavilion 8258, Chicago, IL 60611 USA

Recently we developed stable transfectants of MDA-MB-231 ER-negative breast cancer cells with the cDNA of wild-type (S30 cells) and codon $351_{asp\rightarrow tyr}$ mutant ER (BC-2 cells) (Jiang and Jordan, 1992; Catherino et al, 1995). The naturally occurring codon $351_{asp\rightarrow tyr}$ point mutation in the ligand-binding domain (LBD) of ER was identified in a tamoxifen-stimulated tumour line developed from MCF-7 breast cancer cells implanted into athymic nude mice (Wolf and Jordan, 1994*a* and *b*). Our initial goal was to reassert hormonal control over hormone-independent breast cancer cells by transfecting the hER gene into cells lacking this protein (Jiang and Jordan, 1992). During our investigation of the growth control mechanisms in the S30 cell line, we discovered that oestrogen causes an increase in the mRNA of transforming growth factor alpha (TGF-α) (Jeng et al, 1994). The product can be easily measured by Northern blotting because the basal signal is already dramatically amplified in MDA-MB-231 cells.

The aim of this paper is to report progress in new investigations of TGF-α gene regulation by 17β-oestradiol (E2) and antioestrogens in S30 cells (wild-type ER) (Jiang and Jordan, 1992) and in BC-2 cells (codon $351_{asp\rightarrow tyr}$ mutant ER) (Catherino et al, 1995). Both S30 and BC-2 transfectants exhibit an E2 concentration-dependent induction of TGF-α mRNA expression (Levenson et al, 1997). After an initial examination of the effects of keoxifene (raloxifene) on TGF-α mRNA in our transfectants when raloxifene exhibits oestrogen-like effects with mutant ER (BC-2 cells) but not with wild-type ER (S30 cells) (Levenson et al, 1997), we were surprised to find that the potent tamoxifen metabolite 4-hydroxytamoxifen (4-OHT) (Jordan et al, 1977) produced an increase in TGF-α mRNA levels in a concentration-dependent manner in both cell lines. Thus both an oestrogen– and an anti-oestrogen–ER complex produce the same response at the same

**Figure 1** The plasmid map of the TGF-α ERE-luciferase plasmid constructs used in transient transfection experiments gene. The pure antioestrogen ICI 182,780 (Wakeling et al, 1991), in contrast, can block the induction of TGF-α expression by E2 and 4-OHT in both cell lines. The observation that TGF-α mRNA is induced in response to both E2 and 4-OHT in both cell lines provides us with a powerful and unique model system in which to investigate the mechanism of how both E2 and 4-OHT can activate the same gene. As a first step in dissecting the signal transduction pathway of gene regulation, we took the direct approach of studying gene activation by E2 and 4-OHT through putative oestrogen response elements (EREs) located in the promoter region of the TGF-α gene (Saeki et al, 1991). Unlike the consensus ERE, the putative EREs in the TGF-α promoter region were unable to activate a luciferase reporter gene in response to E2 or 4-OHT. These data suggest that an alternative, more complex mechanism must be available for ligands to initiate transcription of the TGF-α gene.

### MATERIALS AND METHODS

### Cell culture

The MDA-MB-231 cell line used was originally obtained from American Type Culture Collection (Rockville, MD, USA), and the clonal cell line (clone 10A) was used for transfection of either wild-type ER cDNA (HEGO, S30 cells) (Jiang and Jordan, 1992) or codon $351_{\text{asp-tyr}}$ mutant ER cDNA (HETO, BC-2 cells) (Catherino et al, 1995). Cells were maintained in phenol red-free minimal essential medium (MEM) containing 5% charcoal-stripped calf serum, penicillin (100 U $ml^{-1}$), streptomycin (100 μg $ml^{-1}$), L-glutamine (2 mM), G-418 (500 μg $ml^{-1}$), bovine insulin (6 ng $ml^{-1}$) and non-essential amino acids (100 mM). All materials were obtained from Gibco BRL, Life Technologies (Gaithersburg, MD, USA). Oestradiol was purchased from Sigma Chemical (St Louis, MO, USA), 4-OHT was a generous gift from Zeneca Pharmaceuticals (Macclesfield, UK) and ICI 182,780 was a generous gift from Dr Alan Wakeling (Zeneca Pharmaceuticals). All compounds were dissolved in 100% ethanol and added to the media in 1:1000 dilution for a final ethanol concentration no greater than 0.2%.

### Northern blot analysis

Northern blot analysis was performed essentially as described previously (Levenson et al, 1997). Briefly, total RNA was isolated from cells after a 24-h treatment with compounds. Twenty micrograms of RNA sample was fractionated in a 1.2% agarose-formaldehyde gel and transferred to a nylon membrane (Hybond-N+; Amersham, Arlington Heights, IL, USA). The membranes were hybridized at 42°C with $^{32}P$-labelled TGF-α probe (plasmid generously provided by Dr Rik Derynck, Genetech, CA, USA). The membranes were then washed and autoradiographed by exposure to Hyperfilm (Amersham) at −80°C with intensifying screens for 1–2 days. The expected 4.8-kb transcript was detected in both cell lines. Subsequently, the blots were stripped and reprobed with β-actin cDNA. The signals were quantitated by phosphorimage analysis (Molecular Dynamics phosphorimager, Image Quant software).

### Semiquantitative reverse transcriptase–polymerase chain reaction (RT-PCR)

One microgram of total RNA isolated from cells as described above was used in a reverse transcription reaction to obtain cDNA using the SuperScript Preamplification System for First Strand cDNA synthesis kit (Gibco BRL).

Oligonucleotide primers were synthesized using published cDNA sequences for TGF-α (Tahara et al, 1995) and β2-microglobulin (β2-M) (Noonan et al, 1990). The primers used in the PCR reactions are as follows:

| Gene | Primer sequences | Sizes of the amplified product (bp) |
|---|---|---|
| TGF-a | 5′-ATGGTCCCCTCGGCTGGACA | 182 |
| | 5′-CTGCAGGTTCCATGGAAGCA | |
| β2-M | 5′-ACCCCCACTGAAAAAGATGA | 120 |
| | 5′-ATCTTCAAACCTCCATGATG | |

The PCR mixture consisted of the two primers (15 μM), deoxynucleotidetriphosphates (200 μM), template cDNA, 10× buffer (Perkin Elmer), 1.0 unit of AmpliTaq DNA polymerase (Perkin Elmer), 1.5 mM magnesium chloride in a total volume of 25 μl. The optimal number of amplification cycles was determined for each PCR product to avoid the plateau phase. Twenty-five PCR cycles were found to be optimal for both gene products. Cycling was performed with a thermal cycler (Gene Amp PCR System 9600, Perkin Elmer) according to the following parameters: denaturation at 94°C for 30 s, annealing at 55°C for 15 s, extension at 72°C for 30 s, followed by a final incubation at 72°C for 5 min. The PCR products were subjected to 8% polyacrylamide gel electrophoresis. The amplified products were visualized by staining with ethidium bromide. When hot PCR was performed, the gel was dried and then exposed to radiographic film for several hours. The signals were quantified by scanning densitometry of the autoradiograms, and TGF-α was normalized against the β2-M signal.

### Western blot analysis

Whole-cell extracts were prepared from cells treated for 24 h with compounds by lysis of cold phosphate-buffered saline (PBS)-washed cells in lysis buffer (NP40-1%, 20 mM Tris-HCl, 150 mM sodium chloride. The protein concentration was measured using

© Cancer Research Campaign 1998

**Figure 2** Concentration-dependent induction of TGF-α mRNA expression in S30 cells (**A**) and BC-2 cells (**B**) treated with 4-OHT. Total RNA was isolated 24 h after treatment as described in Materials and methods. The Northern blots show the TGF-α 4.8-kb message and the corresponding β-actin signal. The bar graph shows the inducible [the ratio of normalized TGF-α mRNA levels in cells treated with 4-OHT to the normalized TGF-α mRNA levels in untreated cells (control)] levels as determined by densitometric analyses

**Figure 3** Induction of TGF-α mRNA expression in MCF-7, MDA-MB-231 and S30 cell lines analysed by semiquantitative RT-PCR as described in Materials and methods. The figure shows the TGF-α signal (182 bp) and the corresponding β2-M (120 bp) used as an internal control. The sources for RNAs were the following: control, cells treated with EtOH vehicle; E2, cells treated with $10^{-9}$ M oestradiol; 4-OHT, cells treated with $10^{-7}$ M 4-hydroxytamoxifen the Bio-Rad Protein Assay kit (Bio-Rad Laboratories, CA, USA) with bovine gamma globulin as a standard. Equal amounts of protein were subjected to an 8% polyacrylamide gel with a 5% stacking gel. After electrophoresis, proteins were transferred to Hybond, enhanced chemiluminescence (ECL) nitro-cellulose membrane (Amersham, Arlington Heights, IL, USA) using a Mini Trans-Blot Electrophoretic Transfer Cell (Bio-Rad Laboratories). Loading equivalence and transfer efficiency were monitored by Coomassie blue staining of the gel. The membrane was blocked overnight in blocking solution containing PBS-Tween and 7% dry milk. The membrane was then incubated with a 1:500 dilution of the anti-ER antibody H222 in PBS-Tween with 10% calf serum for 2 h at room temperature. The H222 antibody was a generous gift from Abbott Laboratories (Abbott Park, IL, USA). After several washing cycles, horseradish peroxidase-conjugated goat anti-rat IgG antibody (1:2500 dilution) was added to the membrane and incubated for another 2 h at room temperature. The ECL Western blot detection reagents (Amersham, Buckinghamshire, UK) were used for visualization. The ECL detected blots were exposed to autoradiography film (Hyperfilm-ECL) for 1–5 min at room temperature.

### Reporter gene constructs

The reporter construct pERE-Luc contains one *Xenopus laevis* vitellogenin A2 ERE (singlet ERE-luciferase), as has been described previously (Catherino and Jordan, 1995).

We used the same pT109 luciferase plasmid (Nordeen, 1988) for constructs containing the putative TGF-α EREs. Oligonucleotides TGF-α1, TGF-α2, and TGF-$\alpha_{doublet}$ (Figure 1), corresponding to the putative TGF-α EREs previously reported by Saeki et al (1991), were synthesized to contain *Hin*dIII sites at each end. The oligonucleotides were annealed, phosphorylated and ligated into the *Hin*dIII site of the pT109 luciferase plasmid (Nordeen, 1988) and transformed to *E. coli* DH5α cells. Individual colonies were chosen for plasmid preparation and restriction digestion to verify the presence of an insert. Plasmids containing inserts were sequenced to verify the correct sequence, orientation and to ensure the insertion of single and not multiple EREs. Two independent luciferase constructs were prepared for each TGF-α1, TGF-α2 and TGF-$\alpha_{doublet}$ EREs.

The plasmid pCMVβ (Clonetech, Palo Alto, CA, USA), which contains the β-galactosidase gene, was used as an internal control for transient transfection efficiency in all experiments.

### Transient transfection and luciferase assay

MCF-7 and BC-2 cells were seeded in six-well plates at $5 \times 10^5$ cells per well in phenol red-free MEM media containing 5% charcoal-stripped calf serum as described above. Twenty-four hours (MCF-7) or 48 h (BC-2) later, the MCF-7 cells were transiently transfected using the calcium phosphate method (Catherino and Jordan, 1995), and the BC-2 cells using a liposome method (Campbell, 1995). Each well of cells was co-transfected with 1.0 μg of the reporter-luciferase construct along with 0.5 μg of the pCMV-β-gal plasmid to normalize the transfection efficiency. After 4–6 h, the transfection mixture was removed and media containing compound(s) was added. As an intra-assay standard, a vitellogenin singlet ERE/luciferase reporter construct was transfected in parallel to serve as a comparison to each TGF-α-luciferase plasmid. Luciferase activity was measured 18–24 h later using a Monolight 2010 luminometer (Analytical Luminescence Laboratory), and β-gal activity was assayed as in Luyten et al (1988). Total luciferase units were divided by the total β-gal units and expressed as a fold increase over the control (untreated = 1). The mean ± s.e. of at least three independent experiments performed in triplicate was graphed as a percentage of the maximum activity achieved with the vitellogenin ERE/luciferase construct.

*© Cancer Research Campaign 1998*

**Figure 4** Concentration-dependent induction of TGF-α mRNA expression by E2 and 4-OHT in S30 cells. Total RNA was isolated 24 h after treatment with compounds as described in Materials and methods. The Northern blot shows the TGF-α 4.8-kb message and the corresponding β-actin signal. The graph shows inducible levels of TGF-α mRNA [ratio of normalized TGF-α mRNA in cells treated with compounds to normalized levels in untreated cells (control)] as determined by densitometric analyses

### RESULTS

### Concentration-dependent induction of TGF-α mRNA by 4-OHT in S30 and BC-2 cell lines

We have previously demonstrated that there is a concentration-dependent induction of TGF-α mRNA by E2 in S30 (wild-type ER) and BC-2 (mutant ER) cells (Levenson et al, 1997). We now examine the action of 4-OHT on the expression of the TGF-α gene in these transfectants. The effect of 4-OHT on the expression of TGF-α mRNA in S30 and BC-2 cells was determined by Northern blot analyses 24 h after the addition of various concentrations of 4-OHT (Figure 2). Figure 2A shows that 4-OHT stimulates accumulation of TGF-α mRNA in S30 cells in a concentration-dependent manner. This agonist activity of the drug was unexpected because we and others had previously linked changes in the pharmacological properties of non-steroidal antioestrogens with mutations of the ER (Mahfoudi et al, 1995; Montano et al, 1996; Levenson et al, 1997). Similar to the effect of 4-OHT seen in S30 cells (wild-type ER), there was a concentration-dependent induction of TGF-α mRNA in BC-2 cells, expressing mutant ER (Figure 2B). These results suggest that mechanisms other than mutation of the ER are responsible for the agonistic effect of 4-OHT on TGF-α expression in these transfectants. Interestingly, the relative amount of TGF-α mRNA induced in BC-2 cells was more than twice that induced in the S30 (Figure 2).

Our attempt to detect TGF-α mRNA in MCF-7 cells using total RNA in Northern blot were not successful because of the low abundance of transcripts. To illustrate the differences in the cell lines, we used semiquantitative RT-PCR to compare the effect of E2 and 4-OHT on TGF-α mRNA expression in MCF-7, MDA-MB-231 and S-30 cells (Figure 3). Our results show that (1) in MCF-7 cells TGF-α mRNA levels were increased by E2 (fivefold) but not by 4-OHT; (2) in MDA-MB-231 cells TGF-α mRNA levels were unaffected by E2 treatment and were modestly reduced by 4-OHT; and (3) in S30 cells TGF-α mRNA levels were increased by both E2 and 4-OHT by 9.5- and fourfold respectively. Although these data should be viewed as semiquantitative only, they do illustrate the differences in relative amounts of TGF-α mRNA expression in different cell lines in response to E2 and 4-OHT. There is an apparent overexpression of TGF-α mRNA in response to both E2 and 4-OHT in S30 cells.

### Regulation by oestradiol and antioestrogen

As both E2 and 4-OHT were able to stimulate TGF-α mRNA in S-30 and BC-2 cells, we decided to compare the potency of these two ligands. We performed Northern blot analyses of TGF-α mRNA expression using total RNAs from S30 cells treated with various concentrations of both compounds on the same membrane (Figure 4). Both ligands had the same effect on TGF-α mRNA levels at concentrations differing by three orders of magnitude ($10^{-9}$ M for E2 and $10^{-6}$ M for 4-OHT), indicating that E2 was more potent. Although 4-OHT acted as an agonist on TGF-α mRNA expression when added to cells alone, the possibility existed that 4-OHT and E2 would compete with each other for the ER to abolish TGF-α induction. However, the combined treatment of cells with E2 and 4-OHT did not alter TGF-α mRNA induction in either S30 or BC-2 cell lines, whereas the pure antioestrogen ICI 182,780 completely inhibited the action of E2 in both cell lines (data not shown).

### Pure antioestrogen ICI 182,780 remains a complete antioestrogen and is able to block E2 and 4-OHT effects on TGF-α mRNA induction

The intriguing observation that the partial antioestrogen 4-OHT acts as a complete agonist in this model system prompted us to study the effect of other antioestrogens. We have recently reported the antagonistic action of raloxifene on TGF-α mRNA induction in S30 (wild-type ER) cells compared with BC-2 cells expressing the mutant ER (Levenson et al, 1997). Here, we expand our investigation and show that in S30 cells raloxifene blocked not only E2 action on the induction of TGF-α mRNA but also 4-OHT action (Figure 5A). Pure antioestrogen ICI 182,780 blocked the action of E2 and 4-OHT in both cell lines as well as the agonistic action of raloxifene in BC-2 cells (Figure 5B and C).

It is not clear whether stable integration of the transfected ER gene into chromosomal DNA might affect and alter the regulation of ER protein expression by E2 and antioestrogens (Levenson and Jordan, 1994). The mechanism of action for pure antioestrogens (Wakeling and Bowler, 1988) is believed to result from the combined ability to reduce steady-state levels of the ER by increasing the turnover of the protein (Gibson et al, 1991; Dauvois et al, 1992) and to inhibit nucleocytoplasmic shuttling of the receptor by blocking its nuclear uptake (Dauvois et al, 1993).

Therefore, it was of interest to examine the regulation of expression of the ER protein by E2 and antioestrogens in ER-transfected cells. Western blot analyses of whole-cell extracts from S30 and BC-2 cells treated with compound(s) for 24 h revealed an expected 66-kDa ER (Figure 6). As seen in Figure 6A and B levels of ER protein were slightly down-regulated by E2, up-regulated by 4-OHT, not much altered by raloxifene and significantly decreased

**Figure 5** Antagonistic effect of raloxifene on induction of TGF-α mRNA by E2 and 4-OHT in S30 cells (**A**) and effects of E2 and antioestrogens, or combinations of compounds on TGF-α mRNA in S30 (**B**) and BC-2 (**C**) cells analysed by Northern blot as described in Materials and methods. Cells were treated with compound(s) for 24 h. The sources for RNAs were the following: control, cells treated with EtOH vehicle; E2, cells treated with $10^{-9}$ M (**A** and **B**) or $10^{-8}$ M (**C**) oestradiol; 4-OHT, cells treated with $10^{-7}$ M 4-hydroxytamoxifen; Ral, cells treated with $10^{-7}$ M raloxifene; ICI, cells treated with $10^{-6}$ M ICI 182,780. The above-mentioned concentrations for each compound were used alone and in combination experiments. β-Actin was used as a loading control

**Figure 6** Ligand-induced regulation of the 66-kDa exogenous ER proteins in S30 (**A**) and BC-2 (**B**) cells analysed by Western blot as described in Materials and methods. Cells were treated with compound(s) for 24 h. Equal amounts of total protein were run in each lane. The blot was probed with the antibody H222. The sources for proteins were the following: control, cells treated with EtOH vehicle; E2, cells treated with $10^{-9}$ M (**A**) or $10^{-8}$ M (**B**) oestradiol; 4-OHT, cells treated with $10^{-7}$ M 4-hydroxytamoxifen; Ral, cells treated with $10^{-7}$ M raloxifene; ICI, cells treated with $10^{-6}$ M ICI 182,780. The above-mentioned concentrations for each compound were used alone and in combination experiments by ICI 182,780 in both cell lines. Pure antioestrogen ICI 182,780 was able to reduce the amount of ER protein in combination experiments, with the exception of raloxifene in BC-2 cells. It appears that regulation of the steady-state level of the ER protein in transfectants by oestrogen and antioestrogens, in general, is under the same control mechanisms as the steady-state level of endogenous ER in MCF-7 cells (Pink and Jordan, 1996).

### Both E2 and 4-OHT do not activate the putative TGF-α EREs in a luciferase reporter plasmid

Although E2 can increase the expression of TGF-α mRNA and can stimulate the production of TGF-α protein in breast cancer cells (Lippman et al, 1976; Salomon et al, 1989*a* and *b*) and E2-induced expression of TGF-α can be blocked by antioestrogens (Murphy and Dotzlaw, 1989), it is not clear whether these effects of oestrogen are direct or indirect on stimulating transcription of the TGF-α gene. It has been suggested that two potential imperfect palindromic ERE-like sequences are present within the human TGF-α 5′-flanking sequence (Saeki et al, 1991).

To investigate the mechanism of how E2 and 4-OHT can both activate the same gene we performed transient transfection experiments of luciferase reporter constructs containing each of the TGF-α EREs separately, and in combination, retaining the 22-bp intervening sequence naturally found in the TGF-α promoter (Figure 1). Initially, we used the MCF-7 cell line and then confirmed our observations using our transfectants. Figure 7A shows the results of transient transfection of these constructs into MCF-7 cells. There was no luciferase activity when cells were transfected with any of the 'TGF-αERE' constructs. Within the same assay a vitellogenin singlet ERE/luciferase reporter construct was used as a standard and was found to be activated by E2. Similar results were obtained in the T47D breast cancer cell line (data not shown). Figure 7B shows the results of transient transfection experiments of BC-2 cells, which are more easily transfectable than S30 cells. These results show that there is no activation of 'TGF-α EREs' by E2 at any concentrations and in fact there was only a very low activation of the singlet vitellogenin ERE construct. These data demonstrate that the putative TGF-α EREs in the promoter region are very weak and not sufficient alone to mediate either the E2 or the 4-OHT (data not shown) signal using our standardized reporter gene construct with a thymidine kinase (Tk) promoter.

### DISCUSSION

Oestrogens are known to regulate the production of growth factors and their receptors in breast cancer (Lippman and Dickson, 1989). It is well known that TGF-α mRNA and protein is induced by oestrogens in responsive breast cancer cells (Bates et al, 1988;

 © Cancer Research Campaign 1998

**Figure 7** The relative luciferase activity (normalized to β-gal activity) of transiently transfected MCF-7 cells (**A**) and BC-2 cells (**B**) treated with a range of E2 concentrations. As an intra-assay standard, a vitellogenin singlet ERE/luciferase reporter construct was transfected in parallel to serve as a comparison to each TGF-α-luciferase plasmid. The results are expressed relative to the luciferase activities for the maximum fold-increase (over the untreated control) that was achieved with the vitellogenin ERE/luciferase construct for each experiment. The maximum fold-increase for the vitellogenin singlet differed between MCF-7 (50-fold) and BC-2 (threefold) and was assigned an arbitrary value of 100% for each cell line. The mean ± s.e.m. of at least three independent experiments performed in triplicate was graphed. –□–, Vitellogenin; –◇–, TGF-α EREs

Dickson et al, 1992). The fact that this induction is mediated through the ER was supported by experiments with antioestrogens, which were able to block the induction caused by oestrogen (Murphy and Dotzlaw, 1989; Noguchi et al, 1993). The mechanism of induction of TGF-α in cells is presumed to be direct, via the classical pathway in which the receptor binds to EREs in the promoter region of the gene as reported by Saeki et al (1991).

In this report, we present the novel observation that endogenous TGF-α gene expression is stimulated by both E2 and 4-OHT in ER-negative breast cancer cells, stably transfected with either the wild-type (S30 cells) or the mutant ER (BC-2 cells). Thus both oestrogen– and antioestrogen–ER complexes produced the same response at the same gene in ER transfectants. These results were unexpected and suprising for two reasons: (1) tamoxifen is an antioestrogen in breast cancer cells with endogenous ER (MCF-7 cells, Figure 3), whereas it acts as an agonist in ER transfectants; (2) this agonistic activity of the drug was predictable with the mutant receptor but not with wild-type ER. Indeed, we have recently reported the antagonistic action of raloxifene on TGF-α mRNA induction in S30 (wild-type ER) cells compared with BC-2 cells (mutant ER) (Levenson et al, 1997). We expanded our observation with raloxifene in this report and showed that raloxifene acted as an antagonist with wild-type ER and was able to block the effects of both E2 and 4-OHT in S30 cells (Figure 5A).

The pure antiestrogen ICI 182,780 was able to block agonistic activities of E2 and 4-OHT in S30 cells and agonistic activities of all three ligands in BC-2 cells, remaining a complete antagonist with both wild-type and mutant ER (Figure 5B and C). The explanation for the selective agonist/antagonist activity of partial antioestrogens in our model system is currently unclear. However, it is well known that the ligand-induced alterations in the conformation of the ER might be sensed by cellular factors (co-activators or/and co-repressors) that can mediate the activation functions of ER (Halachmi et al, 1994; Smith et al, 1997). We think that identification of such accessory proteins may play a critical role in dissecting the signal transduction pathway in ER transfectants.

The observation that both wild-type and mutant ER did mediate the activation of the TGF-α gene in a similar manner suggests that the mutation in the LBD of the receptor does not affect the activation pathway qualitatively, although we noted quantitative differences (Figure 2). The ER level in both cell lines is quite high (BC-2 cells express a higher level of ER than S30) but similar to that in MCF-7 cells (Catherino et al, 1995). We assume that in addition to the differences between transcription factor pools that interact with the ER in MCF-7 cells compared with parental MDA-MB-231, the different levels of ER in these cell lines might be responsible for the more intense induction of TGF-α in BC-2 cells compared with S30 cells (see Figure 2).

© Cancer Research Campaign 1998

We were not able to detect an E2-stimulated response of luciferase activity after transient transfections of the TGF-α ERE/luciferase reporter constructs. There are several explanations that may account for the inability to detect E2-stimulated luciferase expression in our MCF-7 cells. It is known that MCF-7 sublines differ in their degrees of responsiveness to E2 because of different levels of endogenous ER protein (Butler et al, 1986). By manipulating the levels of ER one might be able to get a different response to E2. Recent data by El-Ashry et al (1996) demonstrated a 30-fold induction of chloramphenicol acetyltransferase (CAT) activity by oestrogen in MCF-7 cells supertransfected with a mouse ER expression vector and the putative TGF-α EREs cloned within the heterologous mouse mammary tumour virus (MMTV) promoter. However, in the absence of the exogenous mouse ER, oestrogen was not able to induce significant and reliable levels of CAT activity in MCF-7 cells, neither with its own TGF-α promoter nor with the TGF-α EREs cloned within the MMTV promoter (El-Ashry et al, 1996). Similarly, in our experiments with a reporter plasmid containing the entire promoter region of the TGF-α gene (pTGF-α-2813Luc, generously provided by Dr D Salomon, NIH, Bethesda, MD, USA), we did not observe induction of luciferase activity in oestrogen-treated MCF-7 cells. We did, however, detect very weak transcriptional activation with both E2 and 4-OHT in BC-2 cells, although the results were variable and inconsistent (data not shown). Thus, our results are in agreement with those of El-Ashry et al (1996) in terms of the inability of the TGF-α promoter and TGF-α EREs to mediate a significant response in MCF-7 cells not boosted with exogenous ER. The discrepancy between our results and those of El-Ashry et al (1996) might be due to differences in the transfected cell lines used (they used MCF-7 and Cos-7 cells transfected with mouse ER, whereas we used a different subline of MCF-7 cells not transfected with exogenous ER and MDA-MB-231 transfected with human ER), and/or in the nature of heterologous promoter used in the reporter constructs (they used MMTV, whereas we used Tk). Finally, consistent with our results, Saeki et al (1991) reported that a fragment that just contained the putative ERE-like elements (pTGF-α-370Luc) was very weak and that additional *cis*-acting elements might be involved in amplifying the effects of E2 in MCF-7 cells.

Both E2 and 4-OHT failed to activate putative TGF-α EREs in MCF-7 and BC-2 cells, suggesting that a pathway other than the classical ERE pathway may be contributing to the induction of the TGF-α gene in these cells. Activation of the activating protein-1 (AP-1) mediated pathway by the ER-ligand complex has been reported as an alternative pathway for ER action in breast cancer cells after long-term tamoxifen treatment (Astruc et al, 1995) as well as in other cell lines (Gaub et al, 1990; Philips et al, 1993; Umayahara et al, 1994; Webb et al, 1995). It is possible that as a consequence of transfection of the ER into cells that were initially ER negative, the classical ER-mediated pathway is shifted towards the alternate pathway.

In summary, we have shown that 4-OHT produces oestrogen-like effects on the induction of TGF-α gene expression in ER transfectants. The observation that the TGF-α gene is activated by both E2 and 4-OHT in breast cancer cells is unique, as in the Ishikawa human endometrial carcinoma cell line in which activation of several genes by both E2 and 4-OHT is reported (Albert et al, 1990; Sundstrom et al, 1990; Jamil et al, 1991; Huynh and Pollak, 1993), TGF-α expression is up-regulated by E2 but not by 4-OHT (Gong et al, 1992). We have, therefore, defined a novel system to test the biochemical mechanism whereby an oestrogen– and an antioestrogen–ER complex can induce the same gene in breast cancer cells. The presented data suggest that there are additional factors present in MDA-MB-231 cells that facilitate gene activation by both an oestrogen– and antioestrogen–receptor complex. These factors may allow the antioestrogen–ER complex to be promiscuous if the ER is overexpressed. We are in the process of dissecting this signal transduction pathway that may suggest a mechanism for the target site-specificity of antioestrogens.

### ABBREVIATIONS

ER, oestrogen receptor; LBD, ligand binding domain; ERE, oestrogen response element; TGF-α, transforming growth factor alpha; E2, 17β-oestradiol; 4-OHT, 4-hydroxytamoxifen; AP-1, activating protein-1; CAT, chloramphenicol acetyltransferase; β2-M, β2-microglobulin; Tk, thymidine kinase; MMTV, mouse mammary tumour virus; RT-PCR, reverse transcriptase–polymerase chain reaction; PBS, phosphate–buffered saline.

### ACKNOWLEDGEMENTS

These studies were supported by the NIH Grant CA-56143, Breast Cancer Program Development Grant R21 CA-65764, and the Lynn Sage Breast Cancer Foundation of Northwestern Memorial Hospital, Chicago. We thank Shashikala Tanjore and Henry Muenzner for excellent technical assistance.

### REFERENCES

Albert JL, Sundstrom SA and Lyttle CR (1990) Estrogen regulation of placental alkaline phosphatase gene expression in a human endometrial adenocarcinoma cell line. *Cancer Res* **50**: 3306–3310

Astruc ME, Chabret C, Bali P, Gagne D and Pons M (1995) Prolonged treatment of breast cancer cells with antiestrogens increases the activating protein-1-mediated response: involvement of the estrogen receptor. *Endocrinology* **136**: 824–832

Bates SE, Davidson NE, Valverius EM, Freter CE, Dickson RB, Tam JP, Kudlow JE, Lippman ME and Salomon DS (1988) Expression of transforming growth factor α and its messenger ribonucleic acid in breast cancer: its regulation by estrogen and its possible functional significance. *Mol Endocrinol* **2**: 543–555

Butler WB, Berlinski PJ, Hillman RM, Kelsey WH and Toenniges MM (1986) Relation of *in vitro* properties to tumorigenicity for a series of sublines of the human breast cancer cell line MCF-7. *Cancer Res* **46**: 6339–6348

Campbell ML (1995) Lipofection reagents prepared by a simple ethanol injection technique. *Bio Techniques* **18**: 1027–1032

Catherino WH and Jordan VC (1995) Increasing the number of tandem estrogen response elements increases the estrogenic activity of a tamoxifen analogue. *Cancer Lett* **92**: 39–47

Catherino WH, Wolf DM and Jordan VC (1995) A naturally occurring estrogen receptor mutation results in increased estrogenicity of a tamoxifen analog. *Mol Endocrinol* **9**: 1053–1063

Dauvois S, Danielian PS, White R and Parker MG (1992) The antiestrogen ICI 164, 384 reduces cellular estrogen receptor content by increasing its turnover. *Proc Natl Acad Sci USA* **89**: 4037–4041

Dauvois S, White R and Parker MG (1993) The antiestrogen ICI 182,780 disrupts estrogen receptor nucleocytoplasmic shuttling. *J Cell Sci* **106**: 1377–1388

Dickson RB, Johnson MD, Bano M, Shi E, Kurebayashi J, Ziff B, Martinez-Lacaci I, Amundadottir LT and Lippman ME (1992) Growth factors in breast cancer: mitogenesis to transformation. *J Steroid Biochem Mol Biol* **43**: 69–78

Early Breast Cancer Trialists' Collaborative Group (1992) Systemic treatment of early breast cancer by hormonal, cytotoxic or immune therapy. 133 randomised trials involving 31,000 recurrences and 24,000 deaths among 75,000 women. *Lancet* **339**: 1–15; 71–85

El-Ashry D, Chrysogelos SA, Lippman ME and Kern FG (1996) Estrogen induction of TGF-α is mediated by an estrogen response element composed of two imperfect palindromes. *J Steroid Biochem Mol Biol* **59**: 261–269

© Cancer Research Campaign 1998

Furr BJA and Jordan VC (1984) The pharmacology and clinical uses of tamoxifen. *Pharmacol Ther* **25**: 127–205

Gaub MP, Bellard M, Scheuer I, Chambon P and Sassone CP (1990) Activation of the ovalbumin gene by the estrogen receptor involves the fos–jun complex. *Cell* **63**: 1267–1276

Gibson MK, Nemmers LA, Beckman Jr WC, Davis VL, Curtis SW and Korach KS (1991) The mechanism of ICI 164,384 antiestrogenicity involves rapid loss of estrogen receptor in uterine tissue. *Endocrinology* **129**: 2000–2010

Gong Y, Ballejo G, Murphy LC and Murphy LJ (1992) Differential effects of estrogen and antiestrogens on transforming growth factor gene expression in endometrial adenocarcinoma cells. *Cancer Res* **52**: 1704–1709

Gottardis MM, Robinson SP, Satyaswaroop PG and Jordan VC (1988) Contrasting actions of tamoxifen on endometrial and breast tumor growth in the athymic mouse. *Cancer Res* **48**: 812–815

Halachmi S, Marden E, Martin G, MacKay H, Abbondanza C and Brown M (1994) Estrogen receptor-associated proteins: possible mediators of hormone-induced transcription. *Science* **264**: 1455–1457

Huynh HT and Pollak M (1993) Insulin-like growth factor-I gene expression in the uterus is stimulated by tamoxifen and inhibited by the pure antiestrogen ICI 182,780. *Cancer Res* **53**: 5585–5588

Jamil A, Croxtall JD and White JO (1991) The effect of antiestrogens on cell growth and progesterone receptor concentration in human endometrial cancer cells (Ishikawa). *Mol Endocrinol* **6**: 215–221

Jeng MH, Jiang SY and Jordan VC (1994) Paradoxical regulation of estrogen-dependent growth factor gene expression in estrogen receptor (ER)-negative human breast cancer cells stably expressing ER. *Cancer Lett* **82**: 123–128

Jiang SY and Jordan VC (1992) Growth regulation of estrogen receptor negative breast cancer cells transfected with complementary DNAs for estrogen receptor. *J Natl Cancer Inst* **84**: 580–591

Jordan VC (1996) *Tamoxifen: A Guide for Clinicians and Patients*. PRR: Huntington, New York

Jordan VC, Collins MM, Rowsby L and Prestwich G (1977) A monohydroxylated metabolite of tamoxifen with potent antioestrogenic activity. *J Endocrinol* **75**: 305–316

Jordan VC, Phelps E and Lindgren JU (1987) Effect of antiestrogens on bone in castrated and intact female rats. *Breast Cancer Res Treat* **10**: 31–35

Levenson AS and Jordan VC (1994) Transfection of human estrogen receptor (ER) cDNA into ER-negative mammalian cell lines. *J Steroid Biochem Mol Biol* **51**: 229–239

Levenson AS, Catherino WH and Jordan VC (1997) Estrogenic activity is increased for an antiestrogen by a natural mutation of the estrogen receptor. *J Steroid Biochem Mol Biol* **60**: 261–268

Lippman ME and Dickson RB (1989) Mechanisms of growth control in normal and malignant breast epithelium. *Recent Prog Horm Res* **45**: 383–440

Lippman ME, Bolan G and Huff K (1976) The effects of estrogens and antiestrogens on hormone responsive human breast cancer in long-term tissue culture. *Cancer Res* **36**: 4595–4601

Love RR, Wiebe DA, Newcomb PA, Cameron L, Leventhal H, Jordan VC, Feyzi J and DeMets DL (1991) Effects of tamoxifen on cardiovascular risk factors in postmenopausal women. *Ann Intern Med* **115**: 860–864

Love RR, Mazess RB, Barden HS, Epstein S, Newcomb PA, Jordan VC, Carbone PP and DeMets DL (1992) Effects of tamoxifen on bone mineral density in postmenopausal women with breast cancer. *N Engl J Med* **326**: 852–856

Luyten GPM, Hoogeveen AT and Galjaard H (1988) A fluorescence staining method for the demonstration and measurement of lysosomal enzyme activities in single cells. *J Histochem Cytochem* **33**: 965

Mahfoudi A, Roulet E, Dauvois S, Parker MG and Wahli W (1995) Specific mutations in the estrogen receptor change the properties of an antiestrogen to full agonists. *Proc Natl Acad Sci USA* **92**: 4206–4210

Montano MM, Ekena K, Krueger KD, Keller AL and Katzenellenbogen BS (1996) Human estrogen receptor ligand activity inversion mutants: receptors that interpret antiestrogens as estrogens and estrogens as antiestrogens and discriminate among different antiestrogens. *Mol Endocrinol* **10**: 230–242

Murphy LC and Dotzlaw H (1989) Regulation of transforming growth factor α and transforming growth factor β messenger ribonucleic acid abundance in T-47D human breast cancer cells. *Mol Endocrinol* **3**: 611–617

Noguchi S, Motomura K, Inaji H, Imaoka S and Koyama H (1993) Down-regulation of transforming growth factor-α by tamoxifen in human breast cancer. *Cancer* **72**: 131–136

Noonan KE, Beck C, Holzmayer TA, Chin JE, Wunder JS, Andrulis II, Gazdar AF, Willman CL, Griffith B, Von Hoff DD and Roninson IB (1990) Quantitative analysis of MDR1 (multidrug resistance) gene expression in human tumors by PCR. *Proc Natl Acad Sci USA* **87**: 7160–7164

Nordeen SK (1988) Luciferase reporter gene vectors for analysis of promoters and enhancers. *Bio Techniques* **6**: 454–457

Philips A, Chalbos D and Rochefort H (1993) Estradiol increases and anti-estrogens antagonize the growth factor-induced activator protein-1 activity in MCF-7 breast cancer cells without affecting c-fos and c-jun synthesis. *J Biol* **268**: 14103–14108

Pink JJ and Jordan VC (1996) Models of estrogen receptor regulation by estrogens and antiestrogens in breast cancer cell lines. *Cancer Res* **56**: 2321–2330

Saeki T, Cristiano A, Lynch MJ, Brattain M, Kim N, Normanno N, Kenney N, Ciardiello F and Salomon DS (1991) Regulation by estrogen through the 5′-flanking region of the transforming growth factor α gene. *Mol Endocrinol* **5**: 1955–1963

Salomon DS, Kidwell WR, Kin N, Ciardiello F, Bates SE, Valverius EM, Lippman ME, Dickson RB and Stampfer MR (1989*a*) Modulation by estrogen and growth factors of transforming growth factor α and epidermal growth factor receptor expression in normal and malignant human mammary epithelial cells. *Recent Results Cancer Res* **113**: 57–69

Salomon DS, Ciardiello F, Valverius EM, Saeki T and Kim N (1989*b*) Transforming growth factors in human breast cancer. *Biomed Pharmacother* **43**: 661–667

Smith CL, Nawaz Z and O'Malley BW (1997) Coactivator and corepressor regulation of the agonist/antagonist activity of the mixed antiestrogen, 4-hydroxytamoxifen. *Mol Endocrinol* **11**: 657–666

Sundstrom SA, Komm BS, Xu Q, Boundy V and Lyttle CR (1990) The stimulation of uterine complement component C3 gene expression by antiestrogens. *Endocrinology* **126**: 1449–1456

Tahara M, Tasaka K, Masumoto N, Adachi K, Adachi H, Ikebuchi Y, Kurachi H and Miyake A (1995) Expression of messenger ribonucleic acid for epidermal growth factor (EGF), transforming growth factor-α (TGFα), and EGF receptor in human amnion cells: possible role of TGFα in prostaglandin E2 synthesis and cell proliferation. *J Clin Endocrinol Metab* **80**: 138–146

Tonetti DA and Jordan VC (1995) Possible mechanisms in the emergence of tamoxifen-resistant breast cancer. *Anti-Cancer Drugs* **6**: 498–507

Tonetti DA and Jordan VC (1996*a*) Design of an ideal hormone replacement therapy for women. *Mol Carcinog* **17**: 108–111

Tonetti DA and Jordan VC (1996*b*) Targeted antiestrogens to treat and prevent diseases in women. *Mol Med Today* **2**: 218–223

Umayahara Y, Kawamori R, Watada H, Imano E, Iwama N, Morishima T, Yamasaki Y, Kajimoto Y and Kamada T (1994) Estrogen regulation of the insulin-like growth factor I gene transcription involves AP-1 enhancer. *J Biol Chem* **269**: 16433–16442

Wakeling AE and Bowler J (1988) Novel antiestrogens without partial agonist activity. *J Steroid Biochem* **31**: 645–653

Wakeling AE, Dukes M and Bowler J (1991) A potent specific pure antiestrogen with clinical potential. *Cancer Res* **51**: 3867–3873

Webb P, Lopez GN, Uht RM and Kushner PJ (1995) Tamoxifen activation of the estrogen receptor/AP-1 pathway: potential origin for the cell-specific estrogen-like effects of antiestrogens. *Mol Endocrinol* **9**: 443–456

Wolf DM and Jordan VC (1994*a*) Characterization of tamoxifen stimulated MCF-7 tumor variants grown in athymic mice. *Breast Cancer Res Treat* **31**: 117–127

Wolf DM and Jordan VC (1994*b*) The estrogen receptor from a tamoxifen stimulated MCF-7 tumor variant contains a point mutation in the ligand binding domain. *Breast Cancer Res Treat* **31**: 129–138

Yang NN, Venugopalan M, Hardikar S and Glasebrook A (1996) Identification of an estrogen response element activated by metabolites of 17β-estradiol and raloxifene. *Science* **273**: 1222–1224

© Cancer Research Campaign 1998